# Nationwide survey of radiation therapy in Japan for lung cancer complicated with interstitial lung disease

**DOI:** 10.1093/jrr/rraa018

**Published:** 2020-05-04

**Authors:** Yasuhito Hagiwara, Yuko Nakayama, Shigehiro Kudo, Toyokazu Hayakawa, Naoki Nakamura, Yoshizumi Kitamoto, Shigeo Takahashi, Kayoko Tsujino, Nobuteru Kubo, Yukihisa Tamaki, Yasushi Nagata, The Japan Radiation Oncology Study Group (JROSG) Working Subgroup for Lung and Mediastinal Tumors

**Affiliations:** 1 Department of Radiation Oncology, Yamagata University Faculty of Medicine, Iida-nishi 2-2-2, Yamagata-shi, 990-9585 Yamagata, Japan; 2 Department of Radiation Oncology, National Cancer Center Hospital, Tsukiji 5-1-1, Chuo-ku, 104-045 Tokyo, Japan; 3 Department of Radiation Oncology, Saitama Cancer Center, Komuro 780, Ina-machi, Kitaadachi-gun, 362-0806 Saitama, Japan; 4 Department of Radiology and Radiation Oncology, Kitasato University School of Medicine, Kitazato 1-15-1, Minami-ku, Sagamihara-shi, 252-0373 Kanagawa, Japan; 5 Department of Radiation Oncology, National Cancer Center Hospital East, Kashiwanoha 6-5-1, Kashiwa-shi, 277-8577 Chiba, Japan; 6 Department of Radiation Oncology, National Hospital Organization Takasaki General Medical Center, Takamatsu-cho 36, Takasaki-shi 370-0829 Gunma, Japan; 7 Department of Radiation Oncology, Kagawa University Faculty of Medicine, Ikenobe 1750-1, Kimi-cho, Kita-gun, 761-0793 Kagawa, Japan; 8 Department of Radiation Oncology, Hyogo Cancer Center, Kitaoji-cho 13-70, Akashi-shi, 673-8558 Hyogo, Japan; 9 Department of Radiation Oncology, Gunma University Graduate School of Medicine, Aramaki-machi 4-2, Maebashi-shi, 371-8510 Gunma, Japan; 10 Department of Radiation Oncology, Shimane University Faculty of Medicine, Enya-cho 89-1, Izumo-shi, 693-8501 Shimane, Japan; 11 Department of Radiation Oncology, Hiroshima University, Kasumi 1-2-3, Minami-ku, Hiroshima-shi, 734-8551 Hiroshima, Japan; 12 The Japan Radiation Oncology Study Group (JROSG) Working Subgroup for Lung and Mediastinal Tumors, Higashi-komagata 2-17-8, Sumida-ku, 130-0005 Tokyo, Japan

**Keywords:** Interstitial lung disease, radiation therapy, acute exacerbation, survey in Japan

## Abstract

The purpose of this study was to clarify the opinions of radiation oncologists in Japan regarding treatment for lung cancer complicated with interstitial lung disease (ILD) by a questionnaire survey, and the risk of acute exacerbation (AE) after radiotherapy. Questionnaires were sent to all of the facilities in which radiation therapy is performed for lung cancer in Japan by using the mailing list of the Japanese Society for Radiation Oncology (JASTRO). The questionnaire survey was conducted to clarify who judges the existence of ILD, the indications for radiation therapy in cases of ILD-combined lung cancer, and the ratio of ILD-combined lung cancer in lung cancer patients treated with radiation therapy. Patients with ILD-combined lung cancer who received radiotherapy during the period from April 2014 to March 2015 were retrospectively analysed. Any cases of AE without any other obvious cause were included. ILD confirmation was performed by central radiologists using computed tomography images. A total of 47 facilities responded to the questionnaire. Radiation therapy was an option in cases of ILD-combined lung cancer in 39 (83%) of the facilities. The indication for radiation therapy was based on image findings in 35 (90%) of the 39 facilities in which radiation therapy was acceptable or was a choice in some cases of ILD. The final indication was based on the opinion of the pulmonologist in 29 (74%) of those 39 facilities. In fiscal year 2014, a total of 2128 patients in 38 facilities received chest irradiation. Seventy-eight (3.7%) of those 2128 patients had ILD-combined lung cancer. Sixty-seven patients were included in patient analysis. AE occurred in 5 patients (7.5%), and one of those 5 patients (20.0%) died from radiation-induced AE. The median period from radiotherapy to AE was 4 months (range, 2–7 months). The following four independent risk factors for AE were identified in univariate analysis: non-advanced age (<75 years), increased C-reactive protein level (≥0.3 mg/dl), adjuvant chemotherapy and ≥ Grade 2 radiation pneumonitis. Radiotherapy was an option for lung cancer even in cases with ILD in 83% (39/47) of the facilities in Japan. Seventy-eight (3.7%) of 2128 patients who received radiation therapy for lung cancer had ILD. Radiotherapy for ILD-combined lung cancer may induce AE at a substantial rate and AE can be life-threatening. Minimizing the risk of radiation pneumonitis might enable the risk of AE to be reduced.

## INTRODUCTION

Interstitial lung disease (ILD), especially idiopathic pulmonary fibrosis, is known to be frequently associated with lung cancer [1]. Although there are few reports of radiotherapy for lung cancer complicated with ILD, it is recognized that radiotherapy for lung cancer can be a risk for acute exacerbation (AE) of ILD [2–7]. However, those retrospective studies were performed in a small number of institutions, and it is not clear whether radiation therapy should be performed for cases of lung cancer with ILD. The difficulty in diagnosis of ILD itself [8, 9] is a factor that makes it difficult to judge the indication for radiation therapy. Thus, the judgment of indication depends on each facility.

The Japanese Respiratory Society conducted a nationwide survey on surgical treatment for lung cancer patients with ILD. Computed tomography (CT) findings considered to be ILD were found in 4.2% of the patients who underwent surgery, of whom 9.3% had AE after surgery and 43.9% died [10, 11].

With regard to radiation therapy, it is necessary to clarify the frequency of ILD, the frequency of AE and the risk factors for AE in lung cancer radiation therapy. Therefore, we conducted a nationwide survey through the Japan Radiological Oncology Research Organization (JROSG) Lung and Mediastinal Tumor Committee. The questionnaire survey was conducted to determine the frequency of ILD in cases of lung cancer, the frequency of radiation treatment for patients with lung cancer who have ILD and the criteria used for indication of radiation treatment in such patients. The patient analysis was conducted to determine the risk factors for AE and the mortality rate after radiotherapy for patients with ILD-combined lung cancer. This trial is registered with UMINCTR as UMIN000036846.

## MATERIALS AND METHODS

### Questionnaire survey

A questionnaire survey was distributed to all of the facilities in Japan in which radiation therapy is performed for lung cancer using the mailing list of the Japanese Society for Radiation Oncology (JASTRO). The questionnaire consisted of four major questions concerning (i) who judges the presence or absence of ILD, (ii) whether radiation therapy is performed for lung cancer in cases with ILD, (iii) the criteria of the indication for radiotherapy in cases with ILD, and (iv) the number of lung cancer patients with ILD who were treated with radiation therapy in 2014. The details of the questionnaire are shown in Supplement 1, see online supplementary material.

### Study design of patient analysis

This study was a multi-center retrospective analysis. The inclusion criteria were as follows: (i) ILD-combined lung cancer cases and (ii) cases treated with thoracic radiotherapy. This study was approved by the appropriate institutional review boards and was carried out in accordance with the Declaration of Helsinki. This study was registered with UMINCTR as UMIN000036846.

### Study endpoints

The primary endpoints of this study were onset of AE and overall survival (OS). OS was calculated from the starting date of radiotherapy. Toxicities were evaluated according to National Cancer Institute Common Terminology Criteria for Adverse Events version 4.0.

### Definition of ILD

**Fig. 1.**
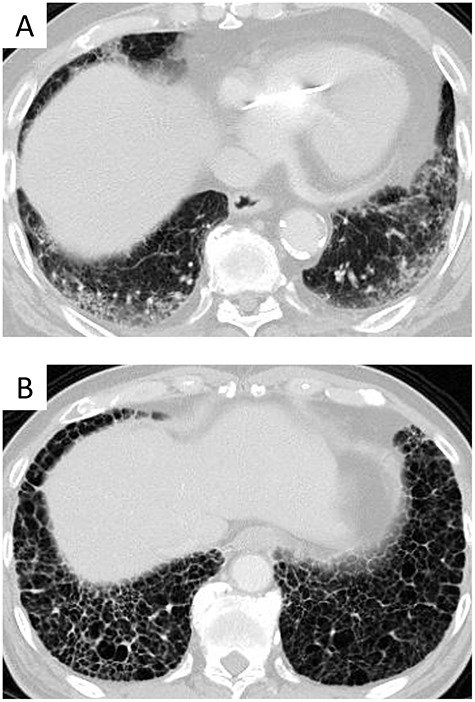
Representative cases of interstitial lung disease. Non-UIP pattern (**A**), and UIP pattern (**B**).

ILD was defined by chest CT as (i) lung disease that has spread from the subpleural part or basal part and (ii) >5% of the whole lung having a reticular shadow, ground glass opacity or accumulation of cysts with clear walls. Patients who fulfilled the two criteria were defined as patients with ILD. ILD was considered to be a usual interstitial pneumonia (UIP) pattern in cases of cysts with clear walls and a non-UIP pattern in other cases. A representative case is shown in Fig. 1. This definition of ILD is based on the Manual for the Diagnosis and Treatment of Idiopathic Interstitial Pneumonia, which was issued in 2004 [12] and is consistent with the European and North American consensus statement [13]. In the Manual for the Diagnosis and Treatment of Idiopathic Interstitial Pneumonia, there is no definition about the proportion of reticular shadow, ground glass opacity or accumulation of cysts with clear walls, in whole lung [12]. We added >5% of the whole lung in definition of ILD, in order to make the judgment more objective.

### Confirmation of ILD diagnosis

CT images were assessed by consultation with two central chest radiologists who were blinded to the clinical and respiratory functional information. Since high-resolution computed tomography (HRCT) was performed in limited patients, confirmation of the ILD diagnosis was dealt with as a supplementary diagnosis. Patients were classified as patients with a UIP pattern of ILD, patients with a non-UIP pattern of ILD and patients with no evidence of ILD.

### Definition of AE after radiotherapy

AE was defined on the basis of criteria proposed by the Japanese Respiratory Society Guideline [12]. These criteria were (i) onset after radiotherapy, (ii) intensified dyspnea within 1 month after onset, (iii) increasing interstitial shadow on a chest radiograph and chest CT scan that had spread through the bilateral lungs including the existing ILD lesion and clearly exceeding the area of the irradiation field, and (iv) no evidence of pulmonary infection, cardiac failure or pulmonary embolism.

### Statistical analysis

The follow-up time was calculated from the starting date of radiotherapy to the last date of follow-up. AE onset and OS were calculated using the Kaplan–Meier method. For univariate analysis, the log-rank test was used to compare AE onset and OS among different subgroups based on patients, lung function and treatment-related factors. For multivariate analysis, Cox proportional hazards regression analysis using variables for which there was a *P*-value < 0.05 in univariate analysis was performed. A *P*-value < 0.05 was considered statistically significant. All statistical analyses were performed using R software, version 3.4.4.

## RESULTS

### Questionnaire survey

Responses to the questionnaire were received from 47 facilities. Thirty-eight of those facilities were JROSG-participating facilities. The response rate from JROSG-participating facilities including 7 particle therapy facilities was 29.2% (38/130 institutions).

#### Judgement of ILD

Judgement of ILD was at the discretion of the respiratory physicians in 41 (87%) of the 47 facilities that responded to the questionnaire. ILD was determined according to the radiologist’s report in 40 (85%) of the 47 facilities. Judgement of ILD was made by radiation oncologists in 37 (79%) of the 47 facilities. Details are shown in Table 1.

**Table 1:** Judgement for interstitial lung disease (number of institutes = 47)

Judged by	Number (%)	Procedure used	Number (%)
Pulmonologist	41 (87%)		
Radiologist	40 (85%)		
Radiation oncologist	37 (79%)	Blood test	28 (76%)
		Physical examination	21 (57%)

#### Radiation therapy for ILD cases

The 47 facilities were divided into three groups with regard to the indication of radiation therapy for ILD cases: acceptable, could be a choice, and unacceptable. Radiation therapy for ILD cases was acceptable in 4 (9%) of the 47 facilities, was a choice in some cases in 35 (74%) of the 47 facilities, and was unacceptable in 8 (17%) of the 47 facilities.

**Fig. 2.**
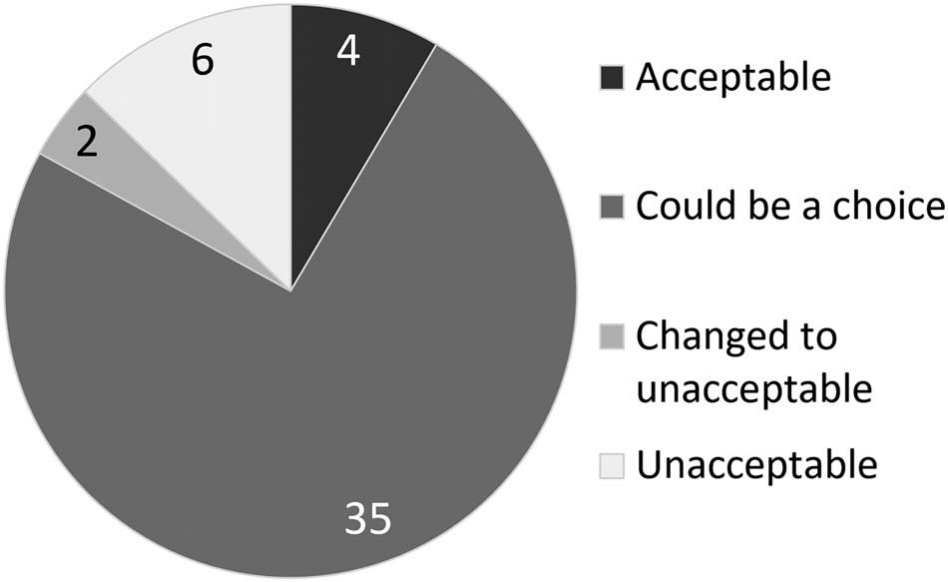
Indication for radiotherapy in cases of lung cancer with interstitial lung disease (number of institutes = 47).

In 2 of the 8 facilities in the unacceptable group, radiation therapy for ILD patients was initially acceptable or was a choice in some cases. Radiation therapy became unacceptable in those 2 facilities because serious life-threatening adverse events occurred. The reasons for radiation therapy being unacceptable in the other 6 facilities were determination by the tumor board, absence of a pulmonologist in the facility, and lack of evidence in guidelines. Details are shown in Fig. 2.

In 7 particle therapy facilities, 2 of the 7 facilities (29%) answered ‘acceptable’ and the other 5 (71%) answered ‘could be a choice’ regarding radiation therapy for cases of lung cancer with ILD. No particle beam treatment facilities answered ‘unacceptable’. In contrast, 5% (2/40) of the photon treatment facilities answered ‘acceptable’, 75% (30/40 facilities) answered ‘could be a choice’ and 20% (8/40 facilities) answered ’unacceptable’ regarding radiation therapy for such cases.

#### Criteria for the indication of radiotherapy in ILD cases

In the 39 facilities in which radiation therapy was acceptable in ILD cases or was a choice in some ILD cases, image findings were the most frequently used criteria for radiotherapy. In 35 (90%) of the 39 facilities, indication for radiation therapy was based on image findings of ILD. In 31 (89%) of those 35 facilities, the proportion of ILD lesions in the lung field was used to determine the indication for radiation therapy.

The second most frequently used criterion was the opinion of the pulmonologist. In 29 (74%) of the 39 facilities, indication for radiation therapy was based on the opinion of the pulmonologist. Results of physical examination were used as criteria in 27 facilities (69%) and results of blood tests were used as criteria in 21 facilities (54%). Cutoff values of Krebs von den lungen-6 (KL-6) and/or surfactant protein D (SP-D) were used in 17 facilities (44%). Details are shown in Table 2.

**Table 2:** Criteria for performing radiotherapy in patients with interstitial lung disease (number of institutes = 39)

Judgement procedure	Details	Number (%)
Image findings		35 (90%)
	Without honeycomb lung	27 (69%)
	Ratio of ILD lesions in lung	31 (79%)
	Without uptake of PET on ILD	14 (36%)
Physical examinations		27 (69%)
	Without HOT	18 (46%)
	Without fine-crackle	13 (33%)
Blood tests		17 (44%)
	KL-6 and SP-D values not exceeding normal values	14 (36%)
	Own limit for KL-6 and SP-D values	7 (18%)
Treatment of ILD		35 (90%)
	Without history of medication for ILD	23 (59%)
	Without ongoing medication with a steroid	25 (64%)
Advice from pulmonologist		29 (74%)
Additional comments		15 (38%)
	No other treatment option	3 (8%)
	Requiring emergency treatment	1 (3%)
	Approval by the tumor board	3 (8%)
	Strongly desired RT even after risk explanation	8 (21%)
	Others	4 (10%)

HOT = home oxygen therapy.

**Table 3:** The number of lung cancer patients with ILD treated with radiation therapy in 2014

Factors	Number of chest RT in fiscal year 2014 Average number of chest RT (range)		
	All institutes 58 (7–226)	<58 chest RT	≥58 chest RT
Number of institutes ILD/chest RT (%)	25/37 (67.6%)	13/22 (59.1%)	12/15 (80.0%)
Number of patients ILD/chest RT (%)	78/2128 (3.7%)	37/726 (5.1%)	41/1402 (2.9%)

RT = radiotherapy.

**Table 4:** Baseline characteristics of patients

Characteristics		Number (%)
Number of patients		67 (100.0)
Sex	Male/female	57 (85.1)/10 (14.9)
Age	Median/range, years	75/57–90
Performance status	0/1/2/3/4	26 (38.8)/28 (41.8)/9 (13.4)/3 (4.5)/1 (1.5)
Brinkman index^a^	Median/range	920/0–4500
Comorbidity	Emphysema	33 (49.3)
	Collagen disease	4 (6.0)
	Chronic heart failure	4 (6.0)
Surgical history of lung	Lobectomy	8 (11.9)
	Segmentectomy	1 (1.5)
	Wedge resection	1 (1.5)
Episode of ILD treatment	Medication	5 (7.5)
	Acute exacerbation before treatment	1 (1.5)
Dyspnea evaluation^b^	%VC median/range	89.2/53.5–131.7
	FEV1% median/range	76.5/49.8–100.0
	Using home oxygen therapy	2 (3.0)
Serum laboratory data	KL-6 (U/I) median/range	559/191–2180
	SP-D (ng/l) median/range	119/7–490
TNM staging^c^	1a	16 (23.9)
	1b	7 (10.4)
	2a	3 (4.5)
	2b	5 (7.5)
	3a	15 (22.4)
	3b	7 (10.4)
	4	14 (20.9)
Histology^d^	Adenocarcinoma	16 (23.9)
	Squamous cell carcinoma	21 (31.3)
	Unclassified non-small cell carcinoma	6 (9.0)
	Small cell carcinoma	
	Clinically diagnosed	10 (14.9)
	Others	11 (16.4)
		3 (4.5)
Radiotherapy		
Type		
Palliative 3D-CRT		14 (20.9)
Definitive 3D-CRT		28 (41.8)
Definitive stereotactic radiotherapy		10 (14.9)
Definitive proton beam therapy		6 (9.0)
Definitive carbon-ion radiotherapy		9 (13.4)
Total dose (Gy (RBE))/fractionation		Median (range)
Palliative 3D-CRT		39 (20–66)/13 (5–33)
Radical 3D-CRT		60 (45–66)/30 (24–50)
Radical stereotactic radiotherapy		48 (48–50)/4 (4–6)
Radical proton beam therapy		66 (60–66)/10 (10–30)
Radical carbon-ion radiotherapy		66 (50–66)/10 (1–10)
Combined chemotherapy	Induction/concomitant/adjuvant	9 (13.4)/16 (23.9)/4 (6.0)

^a^Data were available for 66 patients; ^b^data of %VC and FEV1% were available for 49 and 50 patients, respectively; ^c^stage 4 included 2 patients with postoperative lymph node recurrence; ^d^others included 1 large cell neuroendocrine carcinoma, 1 large cell carcinoma and 1 carcinoma.

FEV1 = forced expiratory volume in 1 s, DLco = diffusion capacity of the lung, TNM = tumor, nodes and metastases, RBE = relative biological effectiveness.

#### The number of lung cancer patients with ILD treated with radiation therapy

We determined the number of cases in which chest radiation therapy for lung cancer (except palliative radiation therapy for spine metastasis) was performed in fiscal year 2014 in 39 institutes in which radiation therapy is an option for lung cancer patients with ILD.

Thirty-seven facilities answered the question, and a total of 2128 patients had received chest irradiation. Seventy-eight (3.7%) of the 2128 patients had ILD-combined lung cancer. The average number of lung cancer patients treated with chest irradiation in each institute was 58 (range 7–226). In 22 of the 37 facilities in which radiation therapy was an option for lung cancer with ILD, lung cancer radiation therapy was performed in fewer than 58 cases each year. In fiscal year 2014, radiation treatment for cases of lung cancer with ILD was performed at 13 institutes (59%). On the other hand, in 15 of the 37 facilities, lung cancer radiation therapy was performed in 58 or more cases each year. In fiscal year 2014, radiotherapy for cases of lung cancer with ILD was performed in 12 (80%) of those 15 facilities. Details are shown in Table 3.

### Patient analysis

#### Baseline characteristics

In total, 78 patients with ILD were identified, and 67 patients were registered in this analysis. The baseline characteristics of the 67 patients are shown in Table 4. Two patients were using home oxygen therapy, 5 patients were receiving medical treatment for ILD and 1 patient had a history of AE prior to radiotherapy. A total of 53 patients received radiotherapy as radical treatment. Fifteen patients received particle-ion radiotherapy.

#### Evaluation of ILD diagnosis

CT images were assessed for 59 of the 67 patients. Fifty-eight (98.3%) of the 59 patients were assessed as having ILD. The judgments of ILD were almost the same by assessment of central chest radiologists and assessment by radiation oncologists. However, the judgments of UIP or non-UIP were the same in only 33 (55.9%) of the 59 patients.

#### Incidence of AE

Five (7.5%) of the 67 patients had AE after radiotherapy. All of the 5 patients recovered from AE, but 1 (20.0%) of the 5 patients had AE relapse without any inducement that resulted in death (Table 5).

**Table 5:** Cases of acute exacerbation

No	Age, years	Sex	TNM stage	Pathology	ILD pattern central/oncologist	CRT	RT technique	Period from RT to AE	History after AE
1	63	Male	cT3N1M0-3a	SqCC	Non-UIP/non-UIP	Yes	3D-CRT 50 Gy/25 fractions	4 months	Recovered from AE, although chemotherapy induced AE again that resulted in death.
2	65	Male	cT2aN2M0-3a	SqCC	UIP/−non-UIP	Yes	3D-CRT 66 Gy/33 fractions	7 months	Recovered from AE. Six months after AE, died from lung cancer.
3	67	Male	cT2aN2M0-3a	NSCLC	non-UIP/non-UIP	Yes	3D-CRT 60 Gy/30 fractions	7 months	Recovered from AE, but AE relapse occurred without any inducement and resulted in death.
4	73	Male	cT3N1M0-2b	SCLC	UIP/non-UIP	Yes	3D-CRT 50 Gy/25 fractions	3 months	Recovered from AE.
5	62	Male	cT1bN0M0-1a	SqCC	UIP/UIP	No	Carbon-ion RT 66 Gy (RBE)/10 fractions	2 months	Recovered from AE. Six months after AE, died from cardiac infarction.

TNM = tumor, nodes and metastases, SqCC = squamous cell carcinoma, NSCLC = non-small cell lung carcinoma, SCLC = small cell lung carcinoma, CRT = chemo-radiotherapy, RT = radiotherapy, 3D-CRT = 3D conformal radiotherapy, RBE = relative biological effectiveness.

### Analysis of prognostic factors

Univariate analysis was used to compare onset of AE and OS among different variables (Table 6). Univariate analysis showed that there was a 6-month period free from AE in larger proportions of patients ≥75 years of age (100.0 vs 90.3%; *P* = 0.0187), patients with C-reactive protein (CRP) < 0.3 mg/dl (100.0 vs 88.6%; *P* = 0.00751), patients who did not receive adjuvant chemotherapy (98.4 vs 50.0%; *P* = 0.000532) and patients with <Grade 2 radiation pneumonitis (98.0 vs 86.2%; *P* = 0.00186). Univariate analysis also showed that there was 6-month OS in larger proportions of patients with white blood cell (WBC) count < 10 000/μl (90.6 vs 44.4%; *P* = 0.000358), patients with CRP < 0.3 mg/dl (96.6 vs 70.7%; *P* = 0.0202), patients with percent vital capacity (%VC) ≥80% (94.1 vs 85.7%; *P* = 0.00569), patients without positron emission tomography (PET) uptake in the ILD lesion (96.3 vs 72.5%; *P* = 0.0494) and patients who received definitive radiotherapy (98.0 vs 100.0%; *P* = 0.000127 × 10^−13^).

**Table 6:** Univariate analysis of different prognostic variables

Prognostic variables	Category	Number of patients	6-month OS (%)	*P* value	6-Month AE-free (%)	*P* value
Age, years	<75	33	81.2	0.377	90.3	0.0187
	≥75	34	85.1		100	
Performance status	0	26	95.8	0.0611	96.2	0.876
	≥1	41	75.4		97.3	
WBC (/μl)	<10,000	54	90.6	0.000358	95.9	0.307
	≥10,000	9	44.4		87.5	
CRP (mg/dl)	<0.3	31	96.6	0.0202	100	0.00751
	≥0.3	31	70.7		88.6	
KL-6 (U/l)	<560	26	84.3	0.328	87.8	0.241
	≥560	23	82.6		100	
SP-D (ng/l)	<120	14	85.7	0.302	100	0.299
	≥120	13	92.3		92.3	
%VC (%)	<80	14	85.7	0.00569	92.9	0.999
	≥80	35	94.1		97.1	
FEV1% (%)	<70	13	100	0.141	100	0.2
	≥70	37	89		94.4	
ILD pattern	Central radiologists	34	76	0.0885	96.7	0.368
	Non-UIP	24	87.1		91.3	
	UIP					
	Radiation oncologists	41	82.5	0.511	94.9	0.399
	Non-UIP	26	74.6		95.7	
	UIP					
PET uptake on ILD	No	27	96.3	0.0494	100	0.222
	Yes	30	72.5		92.2	
Beam type	Photon	52	78.3	0.567	95.6	0.812
	Particle	15	100		93.3	
Purpose	Definitive	53	98	<0.0001	94.3	0.402
	Palliative	14	100	0.000127 × 10^–13^	100	
Radiation-field overlap on ILD	No	30	76.4	0.832	96.3	0.496
	Yes	28	85.4		92.3	
Lung mean (Gy)	<6	32	75	0.89	96.8	0.392
	≥6	34	90.6		93.8	
Dosimetric factors of lung V5 (%)	<22	34	79.4	0.435	97	0.309
	≥22	32	86.7		93.3	
Lung V10 (%)	<16	33	78.8	0.849	96.9	0.345
	≥16	33	87.1		93.5	
Lung V20 (%)	<11	31	73.3	0.875	96.6	0.442
	≥11	34	90.9		94	
Lung V30 (%)	<9	34	76.5	0.207	97	0.318
	≥9	32	90		93.4	
Chemotherapy	No	51	84.2	0.938	95.8	0.514
	Yes	16	81.2		93.8	
Concurrent						
Adjuvant	No	63	83.9	0.905	98.4	0.000532
	Yes	4	75		50	
Radiation pneumonitis	<Grade 2	51	82.1	0.69	98	0.00186
	≥Grade 2	15	86.2		86.2	

FEV1 = forced expiratory volume in 1 s.

Multivariate analysis was carried out using variables that had a *P*-value < 0.05 in univariate analysis (Table 7). Multivariate analysis showed that a larger proportion of patients with ≥Grade 2 radiation pneumonitis had AE onset, though there was no statistically significant association [hazard ratio (HR): 9.2490, 95% confidence interval (CI): 0.7918–108.000, *P* = 0.07609]. However, %VC < 80% was an independent prognostic factor related to OS (HR: 3.38700, 95%CI: 1.074000–10.680, *P* = 0.03737).

**Table 7:** Multivariate analysis of different prognostic variables

	Overall survival			AE-free survival		
Prognostic variables	Hazard ratio	95% CI	*P* value	Hazard ratio	95% CI	*P* value
Age				0.9059	0.7487–1.096	0.30930
WBC (/μl) (<10 000 vs ≥10 000)	1.52200	0.309700–7.483	0.60500			
CRP (mg/dl) (<0.3 vs ≥0.3)	1.73900	0.559300–5.406	0.33910	1.586 × 10^8^	0.0000-infinity	0.99830
%VC (%) (<80 vs ≥80)	3.38700	1.074000–10.680	0.03737			
PET uptake on ILD (no vs yes)	1.86100	0.677500–5.111	0.22830			
Purpose (definitive vs palliative)	0.04863	0.002228–1.061	0.05458			
Adjuvant chemotherapy (no vs yes)				3.7790	0.3839–37.190	0.25450
Radiation pneumonitis (<Grade 2 vs ≥ Grade 2)				9.2490	0.7918–108.000	0.07609

## DISCUSSION

In Japan, lung cancer is usually diagnosed by medical oncologists. Some institutions have tumor boards for patients with lung cancer, and thoracic surgeons, respiratory physicians and radiation oncologists have discussions about which treatment options are acceptable and appropriate for each patient according to the efficacies and the risks of complications [14]. The presentation of accurate risks of complications at a cancer board is important for deciding appropriate treatment options. Nationwide surveys have shown acute exacerbation rates for cases of surgery [10, 11, 15]. However, for radiation therapy, there are only some reports from a limited number of institutes [2–7]. When considering treatment options for lung cancer with ILD, radiation therapy lags behind surgery in risk assessment, and immediate improvement is desired. We therefore carried out this survey to clarify the opinions of radiation oncologists regarding treatment for ILD-combined lung cancer and the risks of acute exacerbation. This report reveals the opinions of radiation oncologists regarding treatment for cases of lung cancer with ILD and how they actually deal with such cases.

In 22 facilities in which lung cancer radiation therapy was performed in fewer than 58 cases in fiscal year 2014, radiation treatment for cases of lung cancer with ILD was performed at 13 institutes (59%). In contrast, in 15 facilities in which lung cancer radiation therapy was performed in 58 or more cases in fiscal 2014, radiotherapy for cases of lung cancer with ILD was performed in 12 (80%) facilities. It seems that in facilities where radiation therapy is performed for a large number of cases every year, radiation therapy is actively performed for cases of lung cancer with ILD. However, when the proportion of cases of lung cancer with ILD in fiscal year 2014 was evaluated in each group, 37/726 cases (5.1%) were cases of lung cancer with ILD in facilities in which lung cancer radiation therapy was performed in <58 cases in fiscal year 2014, whereas 41/1402 cases (2.9%) were cases of lung cancer with ILD in facilities in which lung cancer radiation therapy was performed in ≥58 cases in fiscal year 2014. A comparison of cases of lung cancer with ILD in facilities in which lung cancer radiation therapy was performed in fewer than 58 cases in fiscal year 2014 and in ≥58 cases in fiscal year 2014 showed that facilities that have a large number of lung cancer radiation therapy cases may have a tendency to judge the indication of radiation therapy in ILD cases more carefully. Thus, as an indication for radiation therapy of ILD-combined lung cancer, image findings such as ‘without honeycomb lung’, ‘without uptake of PET on ILD’ and a physical examination such as ‘without fine-crackle’ were dealt with as more important in facilities with a large number of lung cancer radiation therapy cases compared with those facilities with a small number of lung cancer radiation therapy cases. Details are shown in Table 8.

**Table 8:** Difference of criteria for performing radiotherapy in patients with interstitial lung disease according to the number of chest radiotherapies

Judgement procedure	Details	Number of institutes	
		<58 Chest RT	≥58 Chest RT
		22 (100%)	15 (100%)
Image findings			
	Without honeycomb lung	13 (51%)	11 (73%)
	Without uptake of PET on ILD	7 (32%)	6 (40%)
Physical examinations			
	Without fine-crackle	5 (23%)	6 (40%)
Additional comments			
	No other treatment option	0 (0%)	3 (20%)

**Table 9:** Comparison with other series of treatment for ILD-combined lung cancers

Reference	Treatment modality	No. of patients	No. of ILD patients	UIP/non-UIP	AE onset ratio	AE fatality ratio
Sato *et al*. [10]	Surgery	41,742	4.2% (1763/41,742)	73.7% (1300/1,763)/26.3% (463/1,763)	9.3% (164/1 763)	43.9% (72/164)
Kenmotsu *et al*. [19]	Chemotherapy	N/A	N/A (109)	63.3% (69/109)/36.7% (40/109)	22.0% (24/109)	29.2% (7/24)
Yamaguchi *et al*. [20]	SRT	100	16.0% (16/100)	N/A	≥Grade 4 RP 12.5% (2/16)	N/A 50.0% (1/2) was Grade 5 RP
Kim *et al*. [18]	Photon Proton	264	11.4% (30/264)	100.0% (30/30)/0.0% (0/30)	≥Grade 4 RP 18.2% (4/22) 0.0% (0/8)	N/A 75.0% (3/4) died within 1 month after RP
Nakajima *et al*. [16]	carbon-ion	637	4.6% (29/637)	N/A	7.1% (2/28)	0.0% (0/28)
Current study	radiotherapy	2128	3.7% (78/2128)	43.3% (29/67)/56.7% (38/67)	7.5% (5/67) [photon; 7.7% (4/52)]	20.0% (1/5) [photon; 25.0% (1/4)]

N/A = not available, RP = radiation pneumonitis, SRT = stereotactic radiotherapy.

In 39 (83%) of the 47 facilities, radiation therapy for ILD cases was acceptable or was a choice. However, this does not indicate a positive attitude of radiation oncologists toward radiotherapy for cases of ILD-combined lung cancer. Additional comments were given in the questionnaire from 15 (38%) of the 39 facilities regarding restrictions for the indication of radiation therapy for ILD-combined lung cancer cases. Radiation therapy was indicated only for cases with no other treatment option, cases such as stenosis of the trachea requiring emergency treatment, cases in which the patient strongly desired radiation after approval by the cancer board and sufficient explanation to the patient about the risks of treatment, and cases in which informed consent was obtained by more than two physicians. Thus, facilities are trying to provide the best possible treatment with consideration given to the balance of risks and merits.

Seven particle beam treatment facilities were included in this survey. Two of the 7 facilities (29%) answered ‘acceptable’, and no facilities (0%) answered ‘unacceptable’. In contrast, 2 of 40 facilities (5%) of the photon treatment facilities answered ‘acceptable’, and 8 of 40 facilities (20%) answered ‘unacceptable’ regarding radiation therapy for such cases. The opinions regarding radiotherapy for cases of lung cancer with ILD were more positive at particle radiotherapy facilities than at photon therapy facilities. The reason for this difference may be that particle beams are considered to be particularly suitable for high-risk cases due to differences in dose distribution and the social demands associated with it [16–18].

The limitation of this questionnaire survey is that a small number of facilities responded. Of the 47 responses, 38 (81%) were responses from JROSG-participating facilities. However, the response rate from JROSG-participating facilities was limited to 38/130 (29%). The possible reason is that there were limited responses from facilities in which radiotherapy is not performed for cases of lung cancer combined with ILD. In addition, some facilities had few lung cancer patients because there were no pulmonologists or thoracic surgeons in those facilities. In any case, this survey was the first nationwide questionnaire survey on radiation therapy for ILD-combined lung cancer and it has revealed the opinions of Japanese radiation oncologists regarding treatment for lung cancer combined with ILD.

In the patient study, the incidence of AE after radiotherapy was 7.5% (5 of 67 patients) and the mortality rate of AE caused by radiotherapy was 20.0% (1 of 5 patients). Several studies have demonstrated the feasibility of ILD-combined lung cancer treatment using particle-ion radiotherapy [16–18]. However, in this study, there were few differences both in the incidence of AE and the mortality rate even after excluding all patients who received particle-ion radiotherapy: AE onset rate was 7.7% (4 of 52 patients) and mortality rate was 25.0% (1 of 4 patients). The possible reason is that particle-ion radiotherapy institutes have a more positive attitude regarding acceptance of particularly high-risk patients with ILD-combined lung cancer. A comparison with other series of treatment of ILD-combined lung cancer patients is shown in Table 9 [10, 16-17,20–21]. Kim *et al*. reported that 18.2% of patients who received photon radiotherapy showed ≥Grade 4 radiation pneumonitis [18], and Yamaguchi *et al*. reported that 12.5% of patients who received stereotactic radiotherapy showed ≥Grade 4 radiation pneumonitis [20]. However, the results of this study showed that 7.7% of the patients who received photon radiotherapy had AE onset; this included ≥Grade 4 radiation pneumonitis. It should be noted that the results of this study were for strictly selected patients and obtained from limited institutes. This low risk of AE onset was achieved because all of the radiation oncologists concentrated on decreasing the risk of severe radiation-induced toxicities. Similarly, Yamashita *et al*. reported that they selected patients and succeeded in decreasing the risk of ≥Grade 4 radiation pneumonitis from 18.8 to 3.5% [6]. This study suggests that radiotherapy for patients with ILD-combined lung cancer could be acceptable if there is careful patient selection.

In the present study, although there was no statistically significant association in multivariate analysis, patients with ≥Grade 2 radiation pneumonitis tended to have a higher incidence of AE onset (HR: 9.2490, *P* = 0.07609). Based on the relationship between ≥Grade 2 radiation pneumonitis and AE onset, a comparison of the timing of AE onset after surgery and AE onset after radiotherapy showed that there were differences. Those differences arose from the causative events of AE onset. Sato *et al*. reported that the median date of AE onset was 7 days from surgery and that the surgical procedure was a risk factor for AE onset [10]. However, in this study, the median date of AE onset was 4 months from radiotherapy and patients with ≥Grade 2 radiation pneumonitis tended to have a higher incidence of AE onset. Those findings suggest that in surgery, surgery it self and general anesthesia for surgery is the main cause of AE after surgery, whereas radiotherapy, radiotherapy itself is not main cause of AE after radiotherapy. Radiation pneumonitis that occurs a few months after radiotherapy causes AE after radiotherapy. This means that in patients with ILD-combined lung cancer, AE after radiotherapy cannot be separated from ≥Grade 4 radiation pneumonitis.

In a previous study, it was shown that radiotherapy for ILD-combined lung cancer was limited in some institutes’ emergent palliative radiotherapy for patients such as those with stenosis of the trachea. Considering the timing of AE, those strategies were reasonable from the point of view of minimizing the risks and maximizing the merits. Palliative radiotherapy for patients with a poor prognosis could be acceptable.

Sato *et al*. reported a risk scoring system for predicting AE onset after surgery for patients with ILD-combined lung cancer [21]. Surgical procedures (non-wedge resection surgery), history of AE, UIP pattern, male sex, high KL-6 > 1000 U/mL, %VC ≤ 80% and preoperative steroid use were included as risk factors in that scoring system. All of the factors other than the surgical procedure were patient factors. This might mean that the scoring system could be adapted to radiotherapy when high-risk radiotherapy procedures are appropriately defined. In patients with locally advanced lung cancer, Ramella *et al*. reported that if ipsilateral V20 was ≤52% the risk of ≥Grade 2 pneumonitis was 9%, and if ipsilateral V30 was ≤ 39% the risk of ≥Grade 2 pneumonitis was 8% [22]. Chun *et al*. reported that the risk of ≥Grade 3 pneumonitis was low in patients who received intensity-modulated radiation therapy compared to that in patients who received 3D conformal radiotherapy (3D-CRT) (7.9 vs 3.5%) [23]. Hayashi *et al*. reported that the risk of ≥Grade 2 pneumonitis in patients who received carbon-ion radiotherapy for locally advanced lung cancer was 10.6% [24]. In stereotactic radiotherapy, Nagata *et al*. reported that the risk of ≥Grade 3 pneumonitis was 6.5% [25]. From those findings, we defined definitive 3D-CRT as a radiotherapy procedure with a high risk for ≥Grade 2 radiation pneumonitis. The evaluation of risk score is shown in Table 10. The incidences of AE were in good agreement. Those findings suggest that the risk scoring system of AE after surgery might be useful even for radiotherapy.

**Table 10:** Risk score evaluation of acute exacerbation.

Risk group (score)^a^	AE risk of surgery	AE risk of present study (patient number)
Low risk (0–10)	<10%	5.6% (3 AE/54 patients)
Intermediate risk (11–14)	10–25%	16.7% (2 AE/12 patients)
High risk (15–22)	>25%	0.0% (0 AE/1 patients)

^a^Risk score = 5 × (history of AE) + 4 × (surgical procedure other than wedge resection) + 4 × (UIP pattern) + 3 × (male sex) + 3 × (pretreatment steroid use) + 2 × (KL-6 level > 1000 U/ml) + 1 × (%VC < 80%) [21]. In the present study, surgical procedure was replaced with definitive 3D-CRT. Judgment of central radiologists had priority over judgment of UIP pattern by radiation oncologist. In order to avoid an excessively high risk evaluation, variables that were not analysed were dealt with as not applicable.

Although this study was the first nationwide study that was carried out in Japan to determine the frequency of and risk factors for AE and the mortality rate of patients who received radiotherapy for ILD-combined lung cancer, it has several limitations. This study was a retrospective analysis with a limited sample size. The possibility of an influence of additional chemotherapy could not be excluded. Although this study was a nationwide survey, registration of patients was limited to patients in 23 facilities. This might be because the number of institutes in which radiotherapy was actually performed for patients with ILD-combined lung cancer was limited. Particle-ion radiotherapy institutes have a particularly positive attitude regarding radiotherapy for patients with ILD-combined lung cancer, as we reported previously. It is important to clarify to what extent particle-ion radiotherapy is superior to photon radiotherapy. For the next analysis, a well-balanced prospective registry study in which the results of photon radiotherapy and particle-ion radiotherapy are compared is needed to determine which radiotherapy is appropriate in cases of ILD-combined lung cancer.

## CONCLUSIONS

In Japan, radiotherapy was an option for treatment of lung cancer even in cases with ILD in 83% of facilities. The radio of patients with ILD-combined lung cancer among lung cancer patients who underwent radiation therapy was 3.7% (78/2128).

Radiotherapy for ILD-combined lung cancer may induce AE and AE could be life-threatening. Minimizing the risk of radiation pneumonitis and careful patient selection might enable the risk of AE to be reduced. It might be possible to use the surgical AE risk scoring system for assessing AE risk of radiotherapy by replacing risk surgery with definitive 3D-CRT.

## Supplementary Material

Supplement1_rraa018
