# Corrigendum to: Nationwide survey of radiation therapy in Japan for lung cancer complicated with interstitial lung disease

**DOI:** 10.1093/jrr/rraa070

**Published:** 2020-08-13

**Authors:** Yasuhito Hagiwara, Yuko Nakayama, Shigehiro Kudo, Toyokazu Hayakawa, Naoki Nakamura, Yoshizumi Kitamoto, Shigeo Takahashi, Kayoko Tsujino, Nobuteru Kubo, Yukihisa Tamaki, Yasushi Nagata, The Japan Radiation Oncology Study Group (JROSG) Working Subgroup for Lung and Mediastinal Tumors

**Affiliations:** 1 Department of Radiation Oncology, Yamagata University Faculty of Medicine, Iida-nishi 2-2-2, Yamagata-shi, 990-9585 Yamagata, Japan; 2 Department of Radiation Oncology, National Cancer Center Hospital, Tsukiji 5-1-1, Chuo-ku, 104-045 Tokyo, Japan; 3 Department of Radiation Oncology, Saitama Cancer Center, Komuro 780, Ina-machi, Kitaadachi-gun, 362-0806 Saitama, Japan; 4 Department of Radiology and Radiation Oncology, Kitasato University School of Medicine, Kitazato 1-15-1, Minami-ku, Sagamihara-shi, 252-0373 Kanagawa, Japan; 5 Department of Radiation Oncology, National Cancer Center Hospital East, Kashiwanoha 6-5-1, Kashiwa-shi, 277-8577 Chiba, Japan; 6 Department of Radiation Oncology, National Hospital Organization Takasaki General Medical Center, Takamatsu-cho 36, Takasaki-shi 370-0829 Gunma, Japan; 7 Department of Radiation Oncology, Kagawa University Faculty of Medicine, Ikenobe 1750-1, Kimi-cho, Kita-gun, 761-0793 Kagawa, Japan; 8 Department of Radiation Oncology, Hyogo Cancer Center, Kitaoji-cho 13-70, Akashi-shi, 673-8558 Hyogo, Japan; 9 Department of Radiation Oncology, Gunma University Graduate School of Medicine, Aramaki-machi 4-2, Maebashi-shi, 371-8510 Gunma, Japan; 10 Department of Radiation Oncology, Shimane University Faculty of Medicine, Enya-cho 89-1, Izumo-shi, 693-8501 Shimane, Japan; 11 Department of Radiation Oncology, Hiroshima University, Kasumi 1-2-3, Minami-ku, Hiroshima-shi, 734-8551 Hiroshima, Japan; 12 The Japan Radiation Oncology Study Group (JROSG) Working Subgroup for Lung and Mediastinal Tumors, Higashi-komagata 2-17-8, Sumida-ku, 130-0005 Tokyo, Japan

In the Acknowledgement Section, the author wrote ``Hyogo Prefectural Particle Beam Medical Care Center'', but it should be ``Hyogo Ion Beam Medical Center''. This has now been corrected. The author apologizes for this error.

