# Supplementary material for: Nationwide survey of radiation therapy in Japan for lung cancer complicated with interstitial lung disease
Source: J Radiat Res. 2020 May 4;61(4):563–74. doi: 10.1093/jrr/rraa018 (PMC7336568; doi:10.1093/jrr/rraa018)
Supplement: Supplement1_rraa018 [file supplement1_rraa018.docx]

**Supplemental 1:**

Questionnaire that was translated into English.

Below is a questionnaire about thoracic radiotherapy for lung cancer in your institution (except palliative radiotherapy for spinal metastases).

Q1. How do you judge the presence of interstitial lung disease (ILD)?

(check all that apply)

[ ] Has been introduced to your hospital as a case of ILD.

[ ] Respiratory physicians made a diagnosis of ILD.

[ ] Radiologist reported ILD in a report.

[ ] Radiation oncologist made the judgement.

[ ] Judged by diagnostic imaging such as chest radiograph and/or chest CT.

[ ] Judged by blood test.

[ ] Judged by physical exam.

|  |
| --- |

[ ] Others (any additional comments)

Q2. Do you do radiation therapy even for patients with ILD?

[ ] Yes, we do. --> Please go to Q4.

[ ] Could be a choice (depends on the case). --> Please go to Q4.

[ ] We did in the past but not now. → Please go to Q3.

[ ] No, we never do. → Please go to Q5.

|  |
| --- |

If you have any additional comments.

Q3. Please write down the reason why you stopped doing thoracic radiotherapy for lung cancer in cases with ILD.

|  |
| --- |

(free description)

→ Please go to Q4.

Q4. In cases of ILD, what would you use to evaluate the probability of acceptance of radiotherapy?

(check all that apply)

[ ] Judged by images.

[ ] Judged by honeycomb lung.

[ ] Judged by ratio of ILD lesions in the whole lung.

|  |
| --- |

[ ] Others (any additional comments)

[ ] Judged by physical exam.

[ ] Judged by fine crackle sounds on auscultation.

[ ] Judged by having home oxygen therapy or not.

|  |
| --- |

[ ] Others (any additional comments)

[ ] Judged by blood test.

[ ] Scores for KL-6 and / or SP-D do not exceed normal limits.

|  |
| --- |

[ ] You have self-decided limits of scores for KL-6 and/or SP-D.

[ ] Others (any additional comments)

|  |
| --- |

[ ] Judged according to the opinion of respiratory physicians.

[ ] Others (free description)

→ Please go to Q5.

|  |
| --- |

Q5. How many patients have received thoracic radiotherapy (except palliative radiotherapy for spine metastases) for lung cancer in the fiscal first year of 2014 (from April 2014 to March 2015).

patients

(In these cases how many patients received ≥40 Gy?) 　　　　 　　　　 patients

Q6. To facilities in which radiotherapy is acceptable even if the patient has ILD. We would like to conduct a survey on the risk of acute exacerbation of ILD in patients receiving thoracic radiotherapy. Could you cooperate with us?

[ ] Yes.

[ ] No.
